# Implementing Psychological Interventions Through Nonspecialist Providers and Telemedicine in High-Income Countries: Qualitative Study from a Multistakeholder Perspective

**DOI:** 10.2196/19271

**Published:** 2020-08-27

**Authors:** Daisy Radha Singla, Sasha Lemberg-Pelly, Andrea Lawson, Nika Zahedi, Tyla Thomas-Jacques, Cindy-Lee Dennis

**Affiliations:** 1 Sinai Health Toronto, ON Canada; 2 Department of Psychiatry University of Toronto Toronto, ON Canada; 3 Division of Social and Behavioural Health Sciences Dalla Lana School of Public Health University of Toronto Toronto, ON Canada; 4 Women's College Hospital Toronto, ON Canada; 5 Department of Psychology University of Toronto Toronto, ON Canada; 6 Lawrence S Bloomberg Faculty of Nursing University of Toronto Toronto, ON Canada; 7 Li Ka Shing Knowledge Institute St Michael's Hospital Toronto, ON Canada

**Keywords:** nonspecialist providers, task sharing, perinatal mental health, perinatal depression, telemedicine, psychological treatments

## Abstract

**Background:**

Task sharing has been used worldwide to improve access to mental health care, where nonspecialist providers—individuals with no formal training in mental health—have been trained to effectively treat perinatal depressive and anxiety symptoms. Little formative research has been conducted to examine relevant barriers and facilitators of nonspecialist providers and the use of telemedicine in treatment service delivery.

**Objective:**

The primary objective of this study was to examine the main barriers and facilitators of nonspecialist provider–delivered psychological treatments for perinatal populations with common mental health disorders, such as depression and anxiety, from a multistakeholder perspective.

**Methods:**

This study took place in Toronto, Canada. In total, 33 in-depth interviews were conducted with multiple stakeholder groups (women with lived experience and their significant others, as well as health and mental health professionals). Qualitative data were quantified to estimate commonly endorsed themes within and across stakeholder groups.

**Results:**

Psychological treatments delivered by nonspecialist providers were considered acceptable by the vast majority of participants (30/33, 90%). Across all stakeholder groups, nurses (20/33, 61%) and midwives (14/33, 42%) were the most commonly endorsed cadre of nonspecialist providers. The majority of stakeholders (32/33, 97%) were amenable to nonspecialist providers delivering psychological treatment via telemedicine (27/33, 82%), although concerns were raised about the ability to establish a therapeutic alliance via telemedicine (16/33, 48%). Empathy was the most desired characteristic of a nonspecialist provider (61%). Patient and patient advocate stakeholders were more likely to emphasize stigma as an important barrier to accessing psychological treatments (7/12, 58%), compared to clinicians (2/9, 22%) and spouses (1/5, 20%). Clinician stakeholders were more likely to emphasize the importance of ensuring nonspecialist providers were trained to deliver psychological treatments (3/9, 33%), compared to other stakeholder groups.

**Conclusions:**

These results can inform the design, implementation, and integration of nonspecialist-delivered interventions via telemedicine for women with perinatal depressive and anxiety symptoms in high-income country contexts.

## Introduction

Depression is the leading cause of disability worldwide [1], with an estimated 10% to 15% of women experiencing depression and anxiety during pregnancy or in the year following childbirth [2-4]. Psychological interventions, including cognitive, behavioral, and interpersonal therapies, are effective in targeting common perinatal mental health issues such as depression and anxiety [5,6] and are preferred by women over pharmacological treatment [7,8]. However, as few as 20% of women with perinatal depression receive evidence-based psychological treatments [9]. The poor dissemination of effective psychological treatments is due, in part, to the limited number of available mental health professionals [10].

Task sharing has been used worldwide to improve access to care, where nonspecialist providers—individuals with no formal training in mental health, who include peers, lay counsellors, midwives, and nurses—have been trained to effectively treat perinatal depressive and anxiety symptoms worldwide [11,12]. The term “task sharing” is appropriate in high-income countries (HICs) when few physicians are available and tasks may be shared with other providers with some supervision or referral to physicians [13]. This concept was derived from the paraprofessional model in the United States and United Kingdom, where substantive evidence demonstrated the comparative effectiveness of paraprofessional and professional specialists, highlighting paraprofessionals as potential effective additions to mental health fields [14,15]. In low- and middle-income countries (LMICs), nonspecialist providers are an important human resource because they are widely available, cost-effective, and have regular, frequent contact with mothers [16,17]. Similarly, in HICs, nonspecialist providers have been successfully trained to address perinatal mental health [18-20] but little formative research has been conducted to determine the relevant barriers and facilitators of nonspecialist-delivered mental health interventions in HIC contexts.

Telemedicine, the remote delivery of health care services using telecommunication technology that typically involves an audiovisual interface, has become increasingly common among mental health specialists to increase access to health care treatments among perinatal populations [21]. Within high-income countries, psychological treatments delivered via telemedicine have been used to address perinatal depression and have shown great potential for effectively alleviating symptoms [21]. However, the application of telemedicine or other technological solutions in nonspecialist provider–delivered psychological treatments has been largely unexplored [11], despite its potential scalability and widespread use during the COVID-19 pandemic [22]. Exploring these innovations from a multistakeholder perspective may address relevant barriers and facilitators related to the acceptability and demand of mental health services [23,24]. Examining these processes can ultimately facilitate their successful implementation and uptake [24-26].

The primary objective of this study was to examine relevant barriers and facilitators of nonspecialist provider–delivered psychological treatments for perinatal populations with common mental health disorders such as depression and anxiety from a multistakeholder perspective. This included women with lived experience, their significant others, patient advocates, and health care providers such as nurses, midwives, obstetricians, physicians, psychiatrists, psychologists, and health administrators.

Specifically, we aimed to obtain a multistakeholder perspective on the following central research questions:

1. What are the main barriers to and facilitators for perinatal women accessing psychological treatments?
2. Can nonspecialist providers deliver brief psychological treatments for common perinatal mental health issues? If so, who is the preferred nonspecialist provider for psychological treatments in high-income countries? What are their most preferred characteristics?
3. What are the main barriers to and facilitators of telemedicine-delivered psychological treatments for perinatal women when delivered by nonspecialist providers?
4. What is the role of experts in nonspecialist provider–delivered treatments for perinatal women?
5. How can nonspecialist provider–delivered psychological treatments be optimally integrated and sustained within the larger health care system?

## Methods

### Setting and Ethics

The study was conducted at Mount Sinai Hospital in Toronto, Canada. Mount Sinai Hospital is home to Canada’s most productive obstetric and perinatal mental health teams, with almost 10,000 births per year; it is an academic hospital affiliated with the University of Toronto. Ethical approval was obtained from the Research Ethics Board at Mount Sinai Hospital (18-0235-E).

### Participants and Data Collection

Data collection took place between December 2018 and May 2019 through individual, semistructured interviews (see Multimedia Appendix 1 for an example of an interview guide). Key domains included access to psychological treatments during the perinatal period; preferred nonspecialist provider; barriers and facilitators related to nonspecialist providers delivering psychological treatments via telemedicine; the role of experts in nonspecialist-delivered treatments; and integration and sustainability of nonspecialist providers in the broader health care system.

Participants were recruited through an extensive referral network. Perinatal women were first approached by someone in their clinical care team and asked if they could be contacted by a member of the research team regarding an ongoing study. Partners of women were contacted with consent. To avoid response cohesion between partners and spouses, perinatal women were asked to not share interview questions with spouses and research assistants waited a minimum of 2 weeks to contact spouses. Health care professionals, administrators, and mental health specialists were recruited using convenience sampling of the patient and health professional networks and listservs in Toronto.

All consenting individuals were contacted by independent and trained research staff via phone or email and provided information about the study. If individuals agreed to participate, the research staff requested informed consent. Interviews conducted in person took place at Sinai Health System, via phone, or in a confidential location of the participant’s choosing. Two trained interviewers (SL-P and TT-J) conducted and audio-recorded interviews that each lasted 30 to 50 minutes. An index guide was developed to reflect the main barriers and facilitators and these were explored from the perspectives of multiple stakeholders.

### Data Analysis

Content analysis [27] and data analysis (ie, coding) were conducted by 2 independent raters. Using κ, there was a process of first independently coding and then discussing cases until moderate (κ=0.61-0.80) to substantial (κ≤0.81) agreement was achieved. Coding was conducted in a stepwise manner to facilitate iterative revision and then finalization of a coding scheme. Specifically, emergent codes were noted to identify themes in the data. A coding index was developed to organize themes and subthemes. Revisions and additions to codes were made throughout the coding process to most accurately capture themes as they materialized. This process informed the creation of a finalized coding scheme.

After coding, a set of 4 charts (one for each stakeholder group) was created. The charting process was used to organize data into apposite themes and subthemes. Quotations were entered into the charts to provide meaningful exemplars of the themes to which they corresponded. Qualitative data was quantified to reveal the most commonly endorsed themes within stakeholder groups, and then triangulated across stakeholder groups to uncover the most frequently reported themes overall. A numeric table portraying themes with the highest frequencies both within and across stakeholder groups was created to assist with the final analysis.

## Results

### Participant Details

In total, 45 participants were contacted, of which 33 agreed and consented to participate in the study. This included a wide range of stakeholders (see Table 1 for the sampling matrix according to stakeholder group). The majority of interviews (20/33, 60.6%) took place over the telephone as requested by the participant. No consenting participants withdrew from the study.

Coding the qualitative data resulted in a κ value of 0.83*,* indicating substantial agreements between data analysts [28]. Results were triangulated, both within and across stakeholder groups over 5 broad themes that reflected the 5 questions mentioned above: (1) access to psychological treatments during the perinatal period, (2) barriers and facilitators of nonspecialist providers delivering psychological treatments, (3) barriers and facilitators to nonspecialist provider–delivered treatment via telemedicine, (4) the role of experts in nonspecialist provider–delivered treatment, and (5) integrating and sustaining nonspecialist providers within the broader health care system. Results were then used to determine the most commonly endorsed themes overall, and to ascertain similarities and differences in the perspectives of a diverse range of stakeholders. These themes, alongside stakeholder responses, are described in Table 2 and discussed in detail below.

**Table 1:** Participants by stakeholder group (N=33).

Stakeholder			Participants, n
Patients and advocates			12
Clinicians and hospital administrators			9
	Psychiatrists	2	
	Psychologists	1	
	Obstetricians	1	
	Family physicians	3	
	Health administrators	2	
Nurses and midwives			7
Spouses of patients			5

**Table 2:** Key themes related to accessing psychological treatment, accessing nonspecialist provider–delivered treatment, and involving nonspecialist providers in delivering treatment.

Theme			Stakeholder groups								Overall	
			Patients and advocates (n=12), n (%)		Clinicians (n=9), n (%)		Potential nonspecialist providers (n=7), n (%)		Spouses (n=5), n (%)		All stakeholders (N=33), n (%)	
**Access to therapy during the perinatal period**												
	**Barriers**											
		Cost		7 (58)		6 (67)		4 (57)		2 (40)		19 (57)
		Long waitlist times		5 (42)		6 (67)		6 (86)		1 (20)		18 (55)
		Stigma or shame		7 (58)		2 (22)		3 (43)		1 (20)		13 (39)
		Not enough treatment providers		2 (16)		2 (22)		5 (71)		1 (20)		10 (30)
	**Facilitators**											
		Resources on how to find treatment		6 (50)		NE^a^		1 (14)		2 (40)		9 (27)
		Provincial health insurance		2 (20)		3 (33)		2 (29)		1 (20)		8 (24)
		Access to information about mental health		2 (16)		1 (11)		3 (43)		2 (40)		8 (24)
		Training nonspecialists		1 (10)		3 (33)		1 (14)		NE		5 (15)
**Nonspecialist provider–delivered psychological treatment**												
	**Could nonspecialist providers deliver brief talk therapies?**											
		Yes		10 (83)		8 (88)		7 (100)		5 (100)		30 (90)
		No		1 (8)		NE		NE		NE		1 (3)
		Maybe		1 (8)		1 (11)		NE		NE		2 (6)
	**Who would make the ideal nonspecialist provider?**											
		Nurse		9 (75)		5 (55)		5 (71)		1 (20)		20 (61)
		Midwife		3 (25)		3 (33)		5 (71)		3 (60)		14 (42)
	**Most preferred nonspecialist provider characteristics**											
		Empathetic		5 (50)		7 (77)		5 (71)		3 (60)		20 (61)
		Lived experience of mental health issues		7 (58)		NE		3 (43)		4 (80)		14 (42)
		Strong communication skills		3 (30)		6 (66)		2 (29)		1 (20)		12 (36)
		Good listener		4 (33)		2 (22)		5 (71)		1 (20)		12 (36)
	**Barriers**											
		Perceived legitimacy of nonspecialists		2 (16)		2 (22)		4 (57)		1 (20)		9 (27)
		Assessment of symptoms		2 (16)		4 (44)		NE		NE		6 (18)
	**Facilitators**											
		Excellent training and education		8 (67)		3 (33)		4 (57)		3 (60)		18 (55)
		Guidelines for referrals		4 (33)		4 (44)		2 (29)		1 (20)		11 (33)
		Scope of treatment is clearly defined		2 (16)		3 (33)		1 (14)		1 (20)		7 (21)
**Involving nonspecialist providers in delivering treatment**												
	**Barriers**											
		Limited time due to primary profession		3 (25)		NE		3 (43)		1 (20)		7 (21)

^a^NE: no endorsement of theme.

### Theme 1: Access to Psychological Treatments During the Perinatal Period

#### Barriers to Accessing Psychological Treatment

Across all stakeholder groups, the most commonly endorsed barrier to access overall was the cost of therapy (19/33, 57%), with participants noting that psychological treatments are unaffordable and therefore unattainable.

Cost…I think that’s the biggest one [barrier]. And personally, for myself in the past that’s also been like, you know, do I pay rent, or do I prioritize my mental health?

Patient_003

You know when I sought counsel independently, it was $130 an hour. And um during maternal leave… you’re not making any money at all, if you’re being paid for maternity leave maybe 60-80 percent of your wages…so the expense is a barrier as to whether or not you even access that treatment.

Nurse_016

What we also recognize is that once people become aware and are willing to pursue care it is difficult to get timely care...and if you’re depressed post-partum you don’t want to wait. It’s not safe to wait weeks let alone months for care. You want to be seen quickly.

Psychiatrist_009

Alongside cost, reported barriers included long waitlist times (18/33, 55%), stigma (13/33, 39%), and too few treatment providers (10/33, 30%). Among specific stakeholder groups, patients and patient advocates were the most likely to report stigma as the most common barrier to accessing psychological treatments (7/12, 58%).

I think it’s just stigma… after you’ve had a baby and there’s a lot of societal pressure to be really happy…‘well don’t focus on the negative like you should be so happy you should be so grateful that you have a baby a lot of people can’t have babies right?’ So, there was- I felt a lot of shame about going through this.

Patient_017

The stigma attached to it [perinatal anxiety or depression] is a huge barrier.

Advocate_018

#### Facilitators to Accessing Psychological Treatment

Relatedly, and across all stakeholder groups, participants frequently suggested that resources on how to find treatments would be the most common facilitator to improving access (9/33, 27%).

I think it would help people if it was very streamlined to be able to access perinatal mental health care. So, if we were able to for example have one number that everybody in the province could call…or you know, one website…so just making it easier for people to know the right place to go.

Midwife_015

If there was this resource that I hear about just within googling or searching from pregnancy groups…then it would just be a phone call away…or a website away, or a live chat line type of thing then it would be so much easier.

Patient_022

In addition, and across all stakeholder groups, access to information about mental health (8/33, 24%) and provincial health insurance (8/33, 24%) were equally endorsed as the second most common facilitators.

More education around mental health issues during the perinatal period, so people kind of normalizing it for people that this happens. And yeah I guess education about how to go- like letting family doctors know what to look out for and asking the right questions.

Nurse_011

We need to look at mental health and mental and emotional well-being the way we begun to look at physical well-being and health… the media is filled with information about better diet, about exercise…so we need to make mental health and mental illness part of the conversation.

Psychiatrist_009

Within stakeholder groups, some differences emerged. This was especially apparent among mental health clinicians and health administrators who were the only stakeholder group to commonly endorse training nonspecialist providers as a facilitator of access (3/9, 33%).

Use lay therapists…there can be many trained, there are many more of them. The second thing is they [nonspecialist providers] are going to be way less expensive.

Psychiatrist_006

### Theme 2: Barriers and Facilitators of Nonspecialist Providers Delivering Psychological Treatments

#### Overview

The vast majority of participants within and across stakeholder groups answered “yes” to the idea of nonspecialist providers delivering brief psychological treatments (30/33, 90%). When asked what would be the most important nonspecialist provider characteristics, the most frequent responses included having empathy (20/33, 61%), having lived experience with depressive and anxiety symptoms (14/33, 42%), having strong communication skills (12/33, 36%), and being a good listener (12/33, 36%). The majority of stakeholders also reported that both nurses (20/33, 61%) and midwives (14/33, 42%) would be the most acceptable nonspecialist providers to deliver psychological treatments because they work in close contact with perinatal women.

Nurses are so unique because we do have that bio-psycho-social. We know about growth and development, we know about medications, we know about social support and the different things that you can do to make a difference. And I think that the scope of nursing practice I really think that this is an area that we should be expanding into.

Nurse_002

Midwives…are there throughout your care the entire time, and they’re there I think for six weeks post-delivery just to check-in and make sure he’s latching ok, and you’re feeling ok. So, I think that that… natural caring role would be an easy thing to progress with because you’ve already developed a relationship with them.

Patient_019

Perspectives related to the barriers and facilitators of nonspecialist provider–delivered psychological treatments are detailed below and summarized in Table 2.

#### Barriers to Nonspecialist Provider–Delivered Psychological Treatments

Overall, and among all stakeholder groups, the most common barrier to nonspecialist providers delivering psychological treatments was the perspective that others would not perceive nonspecialist providers as legitimate providers (9/33, 27%). This was followed by difficulty in assessing mental health symptoms (6/33, 18%). However, while at least one individual from all stakeholder groups mentioned that patients may not perceive nonspecialist provider–delivered treatment as being as legitimate as specialist-delivered psychological treatments, only clinician and health administrator stakeholders and a small minority of patients and patient advocates expressed that nonspecialist providers may have trouble assessing symptoms. We should also note that fewer barriers (n=2) than facilitators (n=3) of nonspecialist provider–delivered psychological treatments were mentioned across stakeholder groups.

I think another barrier could potentially be even from the client’s perspective, right? Like: ‘who are you? Why are you the one giving me this service when what I wanted was the psychologist or the PhD right? I don’t know what you have to offer me.’

Nurse_005

I think maybe some people might be wondering if these people [nonspecialist providers], maybe they’re not the best because they’re not medically qualified.

Patient_023

In addition, the most commonly reported barrier to involving nonspecialist providers, across stakeholder groups, was that they would have limited time due to other professional responsibilities (7/33, 21%).

Maybe not everyone will want to do it. Because that’s adding an extra task to them that they might not necessarily want to take on, which you know is understandable as well because they have their plates full.

Patient_026

I think one of the biggest barriers is that many people in our health care system feel like they’re working hard and don’t have a lot else to give. So, I think there’s probably lots of people who might in general be supportive of this but they might feel like they might not necessarily have enough time to do it themselves.

Midwife_015

#### Facilitators of Nonspecialist Provider–Delivered Treatment

The most frequently reported facilitator to nonspecialist provider–delivered psychological treatment was high-quality training and education in mental health (18/33, 55%), followed by guidelines for referring to specialist providers (11/33, 33%). Across most stakeholder groups, training and education was the most commonly reported facilitator; one exception was among mental health clinicians and health administrators who most commonly endorsed guidelines for referring to specialist providers as a facilitator for nonspecialist provider–delivered psychological treatments (4/9, 44%). This stakeholder group was also the only group who emphasized that the scope of treatment for nonspecialist providers should be clearly defined (3/9, 33%).

I feel as though it would be valuable to have individuals [nonspecialist providers] in such a role, to be able to at least have those preliminary conversations. And then if they felt that they could not adequately provide that kind of therapy to the person that they’re speaking to, to maybe be able to refer them to someone who is more professionally trained.

Spouse_028

There has to be some sort of model where there’s a referral possible to people that are more widely trained.

Physician_004

I do think that nonspecialists could be trained to do talk therapy … if it is a very specific treatment that they’re doing. I think that where we run into trouble is when nontherapists start to behave as though they are therapists, and they go off script…but I think that certainly for specific interventions it could work very well.

Physician_027

### Theme 3: Barriers and Facilitators to Nonspecialist Provider–Delivered Treatment via Telemedicine

Common barriers and facilitators related to the theme of nonspecialist provider–delivered psychological treatments via telemedicine are summarized in Table 3 and described below.

**Table 3:** Delivering psychological treatments via telemedicine and the role of experts.

Theme			Stakeholder groups				Overall
			Patient and advocates (n=12), n (%)	Clinicians (n=9), n (%)	Potential nonspecialist providers (n=7), n (%)	Spouses (n=5), n (%)	All stakeholders (N=33), n (%)
**Telemedicine**							
	**Could nonspecialist providers use telemedicine?**						
		Yes	10 (83)	8 (88)	6 (86)	2 (40)	26 (79)
		No	NE^a^	NE	NE	2 (40)	2 (6)
		Maybe	NE	1 (11)	NE	NE	1 (3)
		It depends	2 (16)	NE	1 (14)	NE	3 (9)
	**Barriers**						
		Difficult to establish therapeutic alliance	6 (50)	5 (55)	2 (29)	3 (60)	16 (48)
		Lack of access to technology	2 (17)	4 (44)	3 (41)	1 (20)	10 (30)
	**Facilitators**						
		Provide training on telemedicine	1 (8)	2 (22)	3 (43)	NE	6 (18)
		Ensure patients are aware of telemedicine	3 (25)	NE	NE	NE	3 (9)
	**Benefits of telemedicine**						
		Therapy would feel less daunting	5 (50)	NE	1 (14)	3 (60)	9 (33)
		Help decrease issues of access	1 (8)	2 (22)	2 (29)	1 (20)	6 (18)
**Role of experts**							
	**Ideal supervisors**						
		Psychologists	8 (67)	7 (77)	6 (86)	1 (20)	22 (66)
		Psychiatrists	6 (50)	5 (55)	6 (86)	NE	17 (51)
		Social workers	3 (25)	4 (44)	1 (14)	2 (40)	10 (30)
	**Facilitators**						
		Financial compensation	2 (17)	2 (22)	3 (43)	1 (20)	8 (24)
		Sincere desire or interest	2 (17)	1 (11)	3 (43)	1 (20)	7 (21)
		Regular and scheduled meetings	3 (25)	3 (33)	1 (14)	NE	7 (21)

^a^NE: no endorsement of theme.

#### Barriers to Telemedicine

The most commonly reported barrier to telemedicine-delivered psychological treatment was the perception of the difficulty in establishing a strong therapeutic alliance remotely (16/33, 48%). Some stakeholders also brought up the issue of patient access to technology as a barrier to telemedicine-delivered psychological treatments (10/33, 30%). There were no barriers mentioned by any stakeholder about this platform being used by nonspecialist providers in particular.

I’ve heard patients say it’s difficult to often feel comfortable confiding in somebody that you’re just not in the same room with. It doesn’t feel like the same personal connection.

Nurse_011

I think it would remove a bit of the personal aspect of it right. Like although it’s still a video potentially it’s not someone coming to you in person to make you feel like you know there’s someone there who cares.

Patient_021

I mean not having access to the internet, not having the ability to pay for a computer or something with…some sort of video device.

Spouse_029

I think that barriers for telemedicine in general are people who don’t have access to the technology, you know, lack of internet coverage.

Physician_010

#### Facilitators of Telemedicine

Among all stakeholders, common facilitators were reported around the use of telemedicine to deliver psychological treatments. Stakeholders most frequently commented that training on telemedicine would be an important facilitator (6/33, 18%). Specifically, it was recommended that telemedicine-delivered treatment be addressed within the broader training given to nonspecialist providers. Providing training on telemedicine was reported at the highest frequency within stakeholder groups, in addition to ensuring women are aware of telemedicine as a treatment delivery option, which was most commonly endorsed by patients and patient advocates (3/12, 25%).

Maybe you need an extra hour of training in your curriculum or a couple of hours to discuss the special challenge [of telemedicine].

Psychiatrist_006

Just maybe advertising it [telemedicine], getting it out there, letting people know that it’s available. Cause sometimes when there’s really good resources but people don’t know about it then no point in that resource right?

Patient_022

#### Perceived Benefits of Telemedicine

Although participants were not asked about the ways in which telemedicine could be beneficial, many perceived benefits were reported. Across all stakeholders, the most commonly reported benefit that emerged was that telemedicine would make psychological therapies more acceptable and feasible and address the many barriers that new mothers encounter (9/33, 33%). This was especially endorsed by patients, patient advocates, and spouses.

My wife wasn’t comfortable even leaving the house and leaving our child for 7 months. So, the option to be able to video conference, or call and speak to someone is definitely valuable.

Spouse_028

I think that [telemedicine] would help because I think some people are a bit, especially after you just had a baby it’s overwhelming to have to leave your house for anything for a while, especially if you’re feeling depressed or anxious. So, I do think having access to the resources from home is a really good idea.

Patient_021

The second most commonly reported benefit was that telemedicine could decrease issues regarding access to therapy (6/33, 18%).

I certainly think both telemedicine and internet-based therapies are the way we have to go in a country like Canada, where just, the population is so dispersed and the providers aren’t you know, located where the people need them.

Physician_010

I certainly think it’s [telemedicine] helpful for people that live in rural areas, where they can’t access therapy as easily.

Midwife_033

### Theme 4: Role of Experts in Nonspecialist Provider–Delivered Treatment

Across all stakeholder groups, participants reported that the ideal supervisor for nonspecialist providers would be mental health specialists, specifically psychologists (22/33, 66%) and psychiatrists (17/33, 51%). One exception was social workers, who were commonly endorsed by spouses as ideal nonspecialist provider supervisors (2/5, 40%). Overall, stakeholders believed financial compensation to be the greatest incentive to these groups of specialists to provide supervision to nonspecialist providers (8/33, 24%), followed by regular and scheduled meeting times (7/33, 21%), and that supervisors must have a sincere desire or interest in this role (7/33, 21%).

### Theme 5: Integrating and Sustaining Nonspecialist Providers Within the Broader Health Care System

#### Barriers to Integrating Nonspecialist Providers

The most commonly reported barrier to integrating nonspecialist provider–delivered psychological treatment into existing health services was related to competition and concerns raised by mental health specialists (13/33, 39%). The cost involved in implementing nonspecialist provider–delivered treatment was the second most commonly endorsed (8/33, 24%). These results were similar across stakeholder groups (Table 4).

Whenever you’re putting people in a role where psychologists and physicians have been the only ones allowed to practice a role, it’s seen as competition so then they don’t want to support the non, sort of official training therapists because they don’t want their business taken away.

Nurse_011

It is very hard for human beings to act against their self-interest…even in the caring profession where people care about their patients, people will create all kinds of rationalizations why what they are doing, and they are enjoying, and they are well paid to do should continue and should not be changed.

Psychiatrist_006

It’s going to rock the boat a bit I bet…maybe some professionals might not see the benefit in it. They may kind of cringe, like: ’Why are you providing treatment? You’re not professionally trained.’ You know, there’s going to be some backlash I assume and some criticism of nonprofessional providers. So that would kind of be one barrier.

Patient_026

**Table 4:** Integrating and sustaining nonspecialist providers within the existing health care system.

Theme			Stakeholder groups								Overall	
			Patients and advocates (n=12), n (%)		Clinicians (n=9), n (%)		Potential nonspecialist providers (n=7), n (%)		Spouses (n=5), n (%)		All stakeholders (N=33), n (%)	
**Integrating nonspecialist providers**												
	**Barriers**											
		Specialist provider resistance and concerns		5 (42)		4 (44)		3 (43)		1 (20)		13 (39)
		Cost		3 (25)		3 (33)		1 (14)		1 (20)		8 (24)
	**Facilitators**											
		Integrate nonspecialist providers into hospitals or clinics		6 (50)		5 (55)		4 (57)		1 (20)		16 (48)
		Educate patients and providers on nonspecialist providers		4 (33)		1 (11)		3 (43)		2 (40)		10 (30)
		Triage patients into specialist and nonspecialist streams		2 (17)		4 (44)		2 (29)		NE^a^		8 (24)

^a^NE: no endorsement of theme.

#### Facilitators to Integrating Nonspecialist Providers

The most commonly suggested facilitator to integrating nonspecialist provider–delivered psychological treatment reflected the location—in particular, the hospital or primary care practice settings that perinatal populations visit, such as obstetrical units (16/33, 48%). The second most commonly endorsed facilitator focused on educating women and providers about the role of nonspecialist providers in implementing psychological treatments (10/33, 30%).

If it [nonspecialist provider–delivered treatment] could be implemented within a general practice or obstetrician’s office, that person [nonspecialist provider] could become part of the team then that could decrease the cost.

Nurse_016

I guess working as a team with the medical professionals … would be a good start.

Patient_023

One addition to these themes was a suggestion made by many clinician and health administrator stakeholders, who suggested that patients should be triaged into nonspecialist and specialist streams (4/9, 44%).

Look at this as one big team …and that having access so that people are triaged with regard to what their needs are, they can be directed appropriately.

Psychiatrist_009

#### Sustaining Nonspecialist Providers

When asked about the best way to sustain nonspecialist providers in their potential roles to deliver psychological treatments, the most commonly reported answer reflected strategies to avoid burnout (6/16, 38%). This included 4/9 clinicians (44%) and 2/7 potential nonspecialist providers (29%). Patients, patient advocates, and spouses were not queried on this topic.

I think a lot of a turnover in mental health can be because it is a really hard thing to do… compassion fatigue. So, I think a lot of people end up needing to take breaks when they’re providing mental health supports, because it becomes overwhelming for their own mental health.

Midwife_031

Access to supervision or consultation so that they don’t get burnt out or overwhelmed in their role.

Psychologist_001

## Discussion

### Overview

The primary objective of this study was to gather a multistakeholder perspective of the main barriers and facilitators of nonspecialist provider–delivered psychological treatments for perinatal populations with common mental health disorders such as depression and anxiety. We asked a range of stakeholders—including women with lived experiences, their significant others, patient advocates, health care providers, mental health specialists, and health administrators—to comment on accessing psychological treatments, nonspecialist-delivered psychological treatments, the role of experts, the use of telemedicine, and the best way to integrate nonspecialist providers within the broader health care system. In short, and in response to poor access to psychological treatments, we found that psychological treatments delivered by nonspecialist providers were considered both acceptable and feasible by the wide majority of stakeholders, with far more facilitators than barriers mentioned overall. We discuss some additional themes in more detail below.

### Access to Psychological Treatments

The most commonly reported barriers to accessing treatments included cost, stigma, long waitlist times, and not enough treatment providers. The most common facilitators to accessing treatments included resources on how to find treatment, access to information on mental health, and health insurance. Many of these barriers and facilitators have been previously studied [29,30] However, despite an emphasis on resources, there is evidence that increasing funding for resources does not necessarily translate into access. For example, Thornicroft and colleagues [31] demonstrated that despite spending 20-fold more of their gross domestic product on mental health care, the spending was not commensurate with access to resources. In Canada and the United States, access to minimally adequate care remains at 20% of the population compared to 5% in low- and middle-income countries. Clearly, the solution goes beyond investing more resources in specialist providers. Instead, researchers and policy makers alike have argued it is essential that systematic barriers are addressed to optimize health care systems more generally, while also making efforts to prevent mental illness specifically by addressing known risk and protective factors for poor mental health [32].

### Nonspecialist Provider–Delivered Psychological Treatments and the Role of Specialist Providers

In general, the majority of all stakeholder groups reported that nonspecialist providers could indeed be trained to deliver psychological treatments. There is now a rich evidence base, particularly from LMICs, that offers compelling evidence that nonspecialist providers can effectively deliver psychological interventions to manage mood and anxiety disorders among perinatal and general populations [11]. This delivery strategy has the potential to address one of the most significant gaps in mental health care (ie, access to evidence-based psychological treatments). Such approaches are increasingly being advocated for in HICs [33], and it is already a widely accepted approach for mental health care globally (WHO, 2013).

Our findings also suggest that respondents believed that specialist providers could play a unique and important role if nonspecialist providers were trained to deliver psychological treatments. These findings are congruent with a recent review of nonspecialist provider–delivered psychological treatments for common mental disorders [11].The review showed that specialists played the following important roles: assessing psychiatric symptoms, providing referrals and medications, as well as overseeing treatment quality through supervision to ensure that the treatments were implemented with high fidelity (a concern commonly raised by expert clinicians in the current study).

An additional concern raised by a few potential nonspecialist providers and patient stakeholders surrounded whether nonspecialists would be able to fulfil this role on top of their already demanding workloads. These concerns were echoed in a similar study that examined a multistakeholder perspective of nonspecialists delivering PRIME (PRogramme for Implementing Mental health carE) within 5 LMICs [34]. However, there is a dearth of implementation research establishing whether the perception that a nonspecialist role imposes an overly burdensome workload is a real barrier, as opposed to one that is only perceived. Future research could contribute by attempting to clarify this distinction, as well as by exploring facilitators of taking on a nonspecialist provider role alongside other professional duties.

### Preferred Type and Characteristics of Nonspecialist Providers

Across a wide variety of stakeholders, the majority reported that nurses and midwives would be the most appropriate nonspecialist providers to be trained to deliver psychological treatments; respondents in the current study commented that these individuals were most likely to interact with women during the perinatal period. These findings complement the overall literature, which shows that nurses followed by midwives were the most common nonspecialist providers to deliver psychological treatments for perinatal populations (Singla et al, unpublished data, 2020). Of 44 randomized controlled trials for perinatal depression or anxiety, the majority (more than 65%) were delivered by nurses and midwives and were found to effectively treat perinatal depressive and anxiety symptoms. Thus, the perception among some stakeholder groups in the current study that nonspecialist providers could not adequately assess symptoms is inaccurate. It is also important to note that these studies were conducted mainly in Australia and the United Kingdom, which include established models of stepped care (defined below).

In addition, our results suggest that respondents valued communication skills, and wanted their nonspecialist providers to demonstrate empathy and be good listeners, as well as have lived experience with depressive and anxiety symptoms; it is worth noting that despite valuing lived experience as a characteristic, peers (women with lived experience) were not commonly endorsed by stakeholders when asked who the ideal nonspecialist provider would be. These findings reflect studies from both HICs and LMICs. In another qualitative study at two separate sites in Pakistan and India, both communication skills and lived experience were ranked the most preferred characteristics, although the preferred nonspecialist provider was different [16]. Similarly, a recent randomized controlled trial in a HIC context showed that trainees with good interpersonal skills had better patient outcomes [35].

### Telemedicine and Scalability

Overall, the majority of participants across all stakeholder groups responded that nonspecialist providers could be trained to deliver psychological treatments remotely via telemedicine and noted the important implications of telemedicine in improving access to mental health care. However, more barriers than facilitators were presented and individuals questioned whether any treatment provider, nonspecialists and specialists alike, would be able to establish a strong therapeutic alliance with a patient via a telemedicine platform. In contrast to this opinion, there is growing evidence that psychological treatments are effective when delivered remotely [36] and some evidence suggests that a strong therapeutic alliance can be developed and maintained in telemedicine treatments [37]. This is an especially important consideration given the skepticism expressed by some patient stakeholders regarding the ability to establish a therapeutic alliance through telemedicine; patient beliefs about psychological treatments and providers themselves can impact the patient-provider relationship and treatment effectiveness [38]. Additional research is required to examine whether the therapeutic alliance has been maintained in telemedicine-delivered psychological treatments when compared to in-person treatments. This unanswered question complements a recent review that identified the importance of examining the benefits and risks of delivering mental health care through technology instead of face-to-face [39]. In light of the current COVID-19 pandemic, these findings are particularly relevant given the rise of telemedicine in existing health care practice [22].

Furthermore, while remote psychological treatment delivery can be efficacious, there has been minimal exploration of the effectiveness of remote modalities in the context of nonspecialist providers [40]; studies that have explored this have adapted a broader approach than the present study has suggested. For instance, a recent narrative review examined how multiple digital platforms have been leveraged in mental health care to assist nonspecialists with training, diagnosis, treatment guidance, supervision, and the integration of services [41]. Future research is needed to gain an in-depth understanding of nonspecialist provider–delivered treatment via telemedicine, the novel barriers and facilitators that may coincide with this distinct interaction, and whether or not it is effective.

### Integrating and Sustaining Nonspecialist Providers

All stakeholder groups indicated that hospitals or relevant specialty care settings, such as obstetrical units, would be the most acceptable and feasible sites within which to integrate nonspecialist providers, while noting advantages, such as the convenience of having all perinatal health services in one setting. However, stakeholders reported that one major barrier to integrating nonspecialist providers could be resistance from mental health specialists. Specifically, participants across all stakeholder groups, including specialist providers such as psychiatrists and psychologists, expressed concerns that specialist clinicians may feel their positions are being threatened or that they would be skeptical of a nonspecialist provider’s ability to provide adequate care. Despite these perspectives, the literature is extant and growing in arguing that increasing the number of specialists will not resolve the treatment gap issue [10]. Future research is required to address professional protectionism in settings where nonspecialist providers and specialists can deliver mental health care treatments to address perinatal mood disorders.

Relatedly, clinician and hospital administrator stakeholders suggested a stepped care model. This approach provides initial treatment to patients at the lowest adequate intensity. Patients are continually monitored and then stepped up to a more intensive level of care if clinically necessary. This process of gradual treatment adjustment is repeated until the patient’s health status is satisfactory [42]. Stepped care models have been successfully implemented by specialists and nonspecialist providers in a widespread initiative across the United Kingdom, known as Increasing Access for Psychological Treatments [43]. This initiative is a demonstrated stepped model of care with two levels: a low-intensity entry step delivered by nonspecialist providers for the majority of patients with mild to moderate symptoms, and a high-intensity step delivered by specialist providers for the minority of patients who have severe symptoms and do not respond to the first step [43]. This approach has the potential to optimize resources and skill level while maximizing efficiency by reducing wait times [44]. Additional research is required to examine scaling up nonspecialist provider–delivered psychological treatments in community settings during the postpartum period.

### Limitations

We note several limitations in our study, including the use of a convenience sample, interviewing only a small sample of spouses, and not including any policy makers. We did not inquire about or account for participants’ demographic information, including their age and ethnic backgrounds, despite the potential role that age and other relevant socioeconomic variables may play in perceived barriers and facilitators to digital solutions such as telemedicine. Furthermore, there is evidence that cultural beliefs can play a significant role in the acceptance of psychological treatments, treatment providers, and treatment delivery models [45]. This may be particularly relevant for the current study population, which identified barriers among other groups who may have reduced health and digital literacy; this may affect help-seeking for common mental health issues. Future studies are encouraged to assess relevant socioeconomic variables among a random subset of participants to facilitate an improved understanding of and solutions for vulnerable populations, including racial and ethnic minority, lower socioeconomic status, and linguistically diverse populations.

A strength of the current study is its rigorous and systematic use of qualitative methods to examine practical questions related to nonspecialist provider–delivered psychological treatments via telemedicine for perinatal populations. Although this study was framed for perinatal populations specifically, the findings are relevant to other nonspecialist provider–delivered psychological treatments that target a broader audience. In addition, we examined both barriers and facilitators within and across stakeholder groups. This approach, also referred to as integrative data analysis [46], allows for the pooling of data from numerous sources to examine information from multiple perspectives.

### Conclusions

In conclusion, the current study examined the innovations of nonspecialist providers and telemedicine from the perspective of a broad group of stakeholders. Our findings suggest that, despite challenges in accessing psychological treatments, training nonspecialist providers and the use of telemedicine would be largely welcomed and likely used within health care settings. This suggests important implications of acceptability and demand to facilitate future nonspecialist provider–delivered psychological treatments for perinatal and broader populations. In summary, these results can inform the design, implementation, and integration of nonspecialist-delivered interventions via telemedicine for women with perinatal depressive and anxiety symptoms in HIC contexts.

## Appendix

Multimedia Appendix 1 Interview guides.

## Abbreviations

HICs: high-income countries
LMICs: low- and middle-income countries
