# Supplementary material for: Implementing Psychological Interventions Through Nonspecialist Providers and Telemedicine in High-Income Countries: Qualitative Study from a Multistakeholder Perspective
Source: JMIR Ment Health. 2020 Aug 27;7(8):e19271. doi: 10.2196/19271 (PMC7484770; doi:10.2196/19271)
Supplement: Multimedia Appendix 1 [file mental_v7i8e19271_app1.docx]

**Appendix A1. Interview Guide for Patients and Spouses**

1. Across Canada, it is difficult for women (and men) during the perinatal period to access therapy.
   1. What, in your own opinion or experience, is the biggest challenge to individuals receiving access to ‘talk’ therapies (also referred to as psychological treatments)?
      1. [probe: Anything else? What about the availability of people to deliver these treatments?]
   2. What would help overcome this challenge?
2. One solution that has been tested around the world—including Canada—is to train non-mental health professionals to deliver brief, evidence-based treatments for depression and anxiety. These are individuals are not psychiatrists or psychologists, and do not have formal training in mental health care.
   1. Do you think that non-specialists can be trained to deliver a brief talk therapies? [expect Yes or No]
      1. what are some challenges we should consider?
      2. what would help overcome these challenges?
3. Another solution may be to deliver treatments remotely. For example, the Ontario Telemedicine Network (OTN) offers remote treatment services to all healthcare providers across Ontario. Do you think that non-specialists can be trained to deliver the treatment via telemedicine (i.e., delivering therapy through a secure, video-based platform from a distance)?
   1. What would help them do this?
   2. What are some barriers we should consider?
4. Should spouses be included in the delivery of treatment?
   1. [if yes]: Why?
   2. [if no]: Why not?
5. In the Toronto, Ontario or Canadian context, who would be the ideal non-specialist provider? [Probe: Anyone else?]
   1. What would be the best way to recruit these individuals?
   2. What would you say are the most important characteristics for this person? [top 5]
   3. What may be some barriers to consider?
6. In the Toronto, Ontario or Canadian context, who is the ideal supervisor for these non-specialist providers? [probe: what about an expert like a psychologist or psychiatrist?]
   1. What is the best way to recruit these individuals?
   2. What would help facilitate supervision?
7. Do you think NSPs can be trained using a digital training platform?
   1. what are some challenges that we should consider?
   2. what would help facilitate the use of a digital training platform?
8. And if successful, non-specialists offer a scalable solution to improving access to psychological treatments.
   1. What would be the best way to integrate this resource into existing health services?
   2. What are some challenges that would need to be considered?

**Appendix A2. Interview Guide for Clinicians, Administrators and Policy Makers**

1. Across Canada, it is difficult for women (and men) during the perinatal period to access therapy.
   1. What, in your own opinion, is the biggest challenge to individuals receiving access to ‘talk’ therapies (also referred to as psychological treatments)?
      1. [probe: Anything else? What about the availability of people to deliver these treatments?]
   2. What would help overcome this challenge?
2. One solution that has been tested around the world—including Canada—is to train non-mental health professionals to delivery brief, evidence-based treatments for depression and anxiety. These are individuals who are not psychiatrists or psychologists, and do not have formal training in mental health care.
   1. Do you think that non-specialists can be trained to deliver a brief talk therapies?

[expect Yes or No]

- - 1. what are some challenges we should consider?
    2. what would help overcome these challenges?

1. Another solution may be to deliver treatments remotely. For example, the Ontario Telemedicine Network (OTN) offers remote treatment services to all healthcare providers across Ontario. Do you think that non-specialists can be trained to deliver the treatment via telemedicine (i.e., delivering therapy through a secure, video-based platform from a distance)?
   1. What would help them do this?
   2. What are some barriers we should consider?
2. Should spouses be included in the delivery of treatments?
   1. [if yes]: Why?
   2. [if no]: Why not?
3. In the Toronto, Ontario or Canadian context, who would be the ideal non-specialist provider for women during the perinatal phase? [Probe: Anyone else?]
   1. What would be the best way to recruit these individuals?
   2. What would you say are the most important characteristics for this person? [top 5]
   3. What may be some barriers to consider?
      1. Turnover can be an issue. How could we sustain the involvement of these individuals once they are trained in psychological treatments?
4. In the Toronto, Ontario or Canadian context, who is the ideal supervisor for these non-specialist providers? [probe: what about an expert like a psychologist or psychiatrist?]
   1. What is the best way to recruit these individuals?
   2. What would help facilitate supervision?
5. Do you think that these non-specialists could eventually be trained to conduct peer supervision (i.e., supervision among non-specialists)?
   1. what are some challenges that we should consider?
   2. what would help facilitate peer-led supervision?
6. Do you think NSPs can be trained using a digital training platform?
   1. what are some challenges that we should consider?
   2. what would help facilitate the use of a digital training platform?
7. If non-specialists are as effective as specialists in delivering brief psychological treatments, what implications do you think that would have for mental health policy?
8. And if successful, non-specialists offer a scalable solution to improving access to psychological treatments.
   1. What would be the best way to integrate this resource into existing health services?
      1. Probe: How could the organizational context or provider enhance uptake?
   2. What are some challenges that would need to be considered?
